# Factors Associated With Suicide Ideation in US Army Soldiers During Deployment in Afghanistan

**DOI:** 10.1001/jamanetworkopen.2019.19935

**Published:** 2020-01-29

**Authors:** Robert J. Ursano, Holly B. Herberman Mash, Ronald C. Kessler, James A. Naifeh, Carol S. Fullerton, Pablo A. Aliaga, Cara M. Stokes, Gary H. Wynn, Tsz Hin Hinz Ng, Hieu M. Dinh, Oscar I. Gonzalez, Alan M. Zaslavsky, Nancy A. Sampson, Tzu-Cheg Kao, Steven G. Heeringa, Matthew K. Nock, Murray B. Stein

**Affiliations:** 1 Center for the Study of Traumatic Stress, Department of Psychiatry, Uniformed Services University of the Health Sciences, Bethesda, Maryland; 2 Department of Health Care Policy, Harvard Medical School, Boston, Massachusetts; 3 Department of Preventive Medicine and Biostatistics, Uniformed Services University of the Health Sciences, Bethesda, Maryland; 4 Institute for Social Research, University of Michigan, Ann Arbor; 5 Department of Psychology, Harvard University, Cambridge, Massachusetts; 6 Department of Psychiatry, University of California, San Diego, La Jolla; 7 Department of Family and Preventive Medicine, University of California, San Diego, La Jolla; 8 VA San Diego Healthcare System, La Jolla, California

## Abstract

**Importance:**

Understanding suicide ideation (SI) during combat deployment can inform prevention and treatment during and after deployment.

**Objective:**

To examine associations of sociodemographic characteristics, lifetime and past-year stressors, and mental disorders with 30-day SI among a representative sample of US Army soldiers deployed in Afghanistan.

**Design, Setting, and Participants:**

In this survey study, soldiers deployed to Afghanistan completed self-administered questionnaires in July 2012. The sample was weighted to represent all 87 032 soldiers serving in Afghanistan. Prevalence of lifetime, past-year, and 30-day SI and mental disorders was determined. Logistic regression analyses examined risk factors associated with SI. Data analyses for this study were conducted between August 2018 and August 2019.

**Main Outcomes and Measures:**

Suicide ideation, lifetime and 12-month stressors, and mental disorders were assessed with questionnaires. Administrative records identified sociodemographic characteristics and suicide attempts.

**Results:**

A total of 3957 soldiers (3473 [weighted 87.5%] male; 2135 [weighted 52.6%] aged ≤29 years) completed self-administered questionnaires during their deployment in Afghanistan. Lifetime, past-year, and 30-day SI prevalence estimates were 11.7%, 3.0%, and 1.9%, respectively. Among soldiers with SI, 44.2% had major depressive disorder (MDD) and 19.3% had posttraumatic stress disorder in the past 30-day period. A series of analyses of the 23 grouped variables potentially associated with SI resulted in a final model of sex; race/ethnicity; lifetime noncombat trauma; past 12-month relationship problems, legal problems, and death or illness of a friend or family member; and MDD. In this final multivariable model, white race/ethnicity (odds ratio [OR], 3.1 [95% CI, 1.8-5.1]), lifetime noncombat trauma (OR, 2.1 [95% CI, 1.1-4.0]), and MDD (past 30 days: OR, 31.8 [95% CI, 15.0-67.7]; before past 30 days: OR, 4.9 [95% CI, 2.5-9.6]) were associated with SI. Among the 85 soldiers with past 30-day SI, from survey administration through 12 months after returning from deployment, 6% (5 participants) had a documented suicide attempt vs 0.14% (6 participants) of the 3872 soldiers without SI.

**Conclusions and Relevance:**

This study suggests that major depressive disorder and noncombat trauma are important factors in identifying SI risk during combat deployment.

## Key Points

**Question:**

What factors are associated with 30-day suicide ideation among US Army soldiers at the midpoint of their deployment in Afghanistan?

**Findings:**

This survey study of 3957 soldiers who completed self-administered questionnaires at middeployment found an estimated prevalence of lifetime, past-year, and 30-day suicide ideation of 11.7%, 3.0%, and 1.9%, respectively. Risk factors associated with 30-day suicide ideation included white race/ethnicity, lifetime noncombat trauma exposure, and past 30-day and lifetime major depressive disorder.

**Meaning:**

These findings suggest that research examining deployment experiences that increase suicide ideation in soldiers with past trauma and major depressive disorder may assist clinicians and leadership in identifying and treating those at increased risk.

## Teaser

This survey study examines the association of sociodemographic characteristics, lifetime and past-year stressors, and mental disorders with 30-day suicide ideation among US Army soldiers during their deployment in Afghanistan.

## Introduction

Much of our knowledge of mental health (MH) and suicide risk among US Army soldiers is based on postdeployment research.^1,2^ The few studies that have directly examined suicide ideation (SI) and mental health disorders (MHDx) during deployment (in theater) primarily provide descriptive information,^3,4,5^ and, to our knowledge, there have been no population-based survey research studies of risk factors associated with suicidal behaviors in theater. Understanding suicide risk during deployment is particularly important given that an analysis of administrative records from 2004 to 2009 found that the suicide rate among active-duty soldiers in theater during the Iraq and Afghanistan wars was significantly higher than that among never-deployed soldiers.^6^ Furthermore, suicide attempt (SA) rates appear to peak around middeployment, approximately during the sixth month of deployment.^7^

Suicide ideation is associated with elevated risk for suicidal behavior and is an indicator of significant distress and an opportunity for intervention.^8^ Mental health problems in theater are likely underrepresented by administrative records, given barriers to treatment seeking among soldiers with MHDx,^9^ and the challenges of medical record keeping in combat environments. Survey research can directly measure undocumented MHDx and SI during deployment; however, logistical hurdles make such studies rare. Understanding of self-reported SI in theater is primarily informed by nonrepresentative survey research conducted by the US military for the purposes of MH surveillance, which found that 7% of soldiers and Marines reported past-month SI during deployment in Afghanistan.^10^ Similar to documented SAs, risk of self-reported SI and other MH symptoms were noted to peak around middeployment.^5,7^

A rapidly growing body of research has identified risk factors associated with SI among service members, including sociodemographic characteristics (eg, being female, younger, older when first entering service, less educated, never married),^11^ traumatic and life stressors (eg, adverse childhood experiences, military sexual trauma, combat exposure),^12,13,14,15,16^ and MHDx (eg, current and lifetime posttraumatic stress disorder [PTSD] and major depressive disorder [MDD], problematic alcohol use).^17,18,19^ However, it is not known whether these factors are differentially associated with SI during deployment, a time when soldiers are exposed to unique stressors and the context of threat may alter established mechanisms of suicide risk. Given the elevated risk of suicidal behaviors in middeployment, identification of risk factors during this period is critical.

We used data from the Army Study to Assess Risk and Resilience in Servicemembers (Army STARRS)^20^ to examine prevalence of self-reported MHDx and factors associated with 30-day SI in a unique, representative sample of soldiers surveyed at the midpoint of their deployment. First, we identified MHDx frequency during deployment. Next, beginning with a broad range of sociodemographic characteristics, lifetime and 12-month stressors, and MHDx, we systematically examined independent influences of individual factors associated with in-theater SI, followed by identification of the best set of multivariable factors in a combined model. We then determined the associated prevalence of documented SAs following survey administration.

## Methods

### Sample

Soldiers on deployment to Afghanistan completed self-administered questionnaires in July 2012 (eMethods in the Supplement) as part of the All Army Study (AAS),^20,21,22^ a representative population survey of active-duty soldiers (Regular Army and activated Army National Guard/Reserve) excluding those in basic training. Data were collected in Kuwait as soldiers were transiting for middeployment leave. Among soldiers recruited for this population study, 80.9% provided informed consent, and 86.5% of these completed the self-administered questionnaire. Of these, 55.6% consented to linkage of their responses to Army and Department of Defense (DoD) administrative records. The final analytic sample of 3957 was weighted to be representative of all 87 032 active-duty soldiers serving in Afghanistan in July 2012. Detailed information regarding the weighting process, which allows for report of population prevalence estimates, is found in eMethods in the Supplement and the article by Kessler et al.^22^ All recruitment, informed consent, and data protection procedures were approved by the Human Subjects Committees of all collaborating organizations. Data analyses were conducted between August 2018 and August 2019. This study followed the Strengthening the Reporting of Observational Studies in Epidemiology (STROBE) reporting guideline.

### Measures

#### Sociodemographic Characteristics

Sociodemographic characteristics included sex, age, race/ethnicity, education, and marital status. These variables were constructed using Army and DoD administrative records.

#### Mental Health Disorders

The self-administered questionnaire included assessment of *Diagnostic and Statistical Manual of Mental Disorders* (Fourth Edition) internalizing and externalizing disorders. The Composite International Diagnostic Interview screening scales^23,24^ assessed past 30-day MDD, generalized anxiety disorder, panic disorder, substance use disorder (alcohol and/or drug abuse or dependence, including illicit drugs and misused prescription drugs), and intermittent explosive disorder (IED) and past 6-month attention-deficit/hyperactivity disorder. Past 30-day PTSD was assessed using the PTSD Checklist.^25^ The Composite International Diagnostic Interview screening scales also assessed lifetime panic disorder, IED, and bipolar disorder I or II or subthreshold bipolar disorder. Subthreshold bipolar disorder was defined as lifetime history of hypomania without MDD or subthreshold hypomania with MDD history.^26^ Lifetime MDD, generalized anxiety disorder, substance use disorder, and PTSD were assessed using a revised self-administered version of the Family History Screen,^27^ measuring personal, rather than family, MHDx history. All disorders were assessed without *Diagnostic and Statistical Manual of Mental Disorders* (Fourth Edition) diagnostic hierarchy or organic exclusion rules. The Composite International Diagnostic Interview screening scales and PTSD Checklist have good concordance with independent clinical diagnoses in the AAS (area under the receiver operating characteristic curve of 0.69-0.79 across diagnoses).^28^ The Family History Screen has acceptable concordance with best-estimate clinical diagnoses,^27^ although items used in the AAS yielded implausibly high prevalence estimates, and Family History Screen diagnoses should consequently be considered combinations of threshold and subthreshold disorders. Data on MHDx were used to construct recency variables (past 30 days, prior to past 30 days, no diagnosis) for MDD, generalized anxiety disorder, panic disorder, PTSD, substance use disorder, and IED. Bipolar disorder was examined only as a lifetime diagnosis (owing to small sample size) and attention-deficit/hyperactivity disorder was examined only as a past 6-month diagnosis (owing to how it was assessed).

#### Stressors

We assessed lifetime and past 12-month exposure to traumatic and stressful events. Using items from the Joint-Mental Health Advisory Team 7^29^ and Deployment Risk and Resilience Inventory,^30^ respondents were asked how many times they had experienced 15 deployment-related stressors (eg, fired rounds at enemy or taken enemy fire, wounded by enemy, unit members seriously wounded or killed, hazed or bullied by unit members) and 14 life stressors excluding deployment experiences (eg, serious physical assault, sexual assault or rape, murder of close friend or relative, life-threatening illness or injury, disaster). Responses to these questions were dichotomized (yes or no). Respondents also indicated (yes or no) whether they had experienced any of the events in the past 12 months. Additional 12-month stressors were assessed (yes or no) using 16 items from the Life Events Questionnaire^31^ and 2008 DoD Survey of Health-Related Behaviors Among Active Duty Military Personnel^32^ (eg, life-threatening illness of close friend or relative, separation or divorce, caused an accident in which someone else was hurt, trouble with police).

#### SI and SA

Past 30-day SI was assessed using a modified version of the Columbia Suicidal Severity Rating Scale.^33^ Respondents endorsing lifetime SI (“Did you ever in your life have thoughts of killing yourself?” or “Did you ever wish you were dead or would go to sleep and never wake up?”) were asked whether SI occurred in the past 30 days and the ages at which they first and last experienced SI. Administratively documented SAs in theater and up to 12 months following return from deployment were identified using DoD Suicide Event Report^34^ records, and *International Classification of Diseases, Ninth Revision, Clinical Modification *diagnostic codes E950 to E958 from the Military Health System Data Repository, Theater Medical Data Store, and TRANSCOM (Transportation Command) Regulating and Command and Control Evacuating System.^35^ Soldiers who endorsed SI in the past 30 days were labeled as cases and those who did not were considered controls.

### Statistical Analysis

Frequencies of MHDx were calculated. Factors associated with past 30-day SI were examined in stages, beginning with univariable associations of sociodemographic variables. Sociodemographic characteristics significant at the univariable level (*P* < .05) were examined together in a multivariable model. One exception was the a priori decision to include sex in subsequent multivariable models because of its consistent association with SI in previous Army^11,36,37,38^ and civilian^39,40,41^ studies. A similar process was used for MHDx variables, except that the multivariable model included significant sociodemographic characteristics from the previous step.

Owing to the small number of cases and large number of stressors, exploratory factor analysis was used as a data reduction method to identify latent stressor subgroups. We conducted a polychoric exploratory factor analysis with promax rotation using the lifetime stressors (30 items), followed by a similar exploratory factor analysis using past 12-month stressors (46 items). For each factor, we constructed dichotomous (any event type vs none) and cumulative (count of event types) variables.

Associations of stressor variables with SI were examined in a series of logistic regression models. The factor-based, dichotomous lifetime stressor variables were examined together in a multivariable model, followed by a separate model that also included the cumulative scores for each lifetime factor. A final lifetime stressor model included all significant cumulative scores, together with any significant dichotomous factors that were not significant at the cumulative level. This procedure was repeated with the factor-based 12-month stressors. Significant stressor variables from the final lifetime and past 12-month stressor models were then included together in a model identifying factors associated with SI. This approach of testing the contribution of an entire variable set with a multiple degrees of freedom test addresses possible correlations among the variables and improves model selection.^42^

Next, significant variables from the sociodemographic, stressor, and MHDx analyses were examined together in combined models. Logistic regression coefficients were exponentiated to obtain odds ratios (ORs) and 95% confidence intervals. Standard errors were estimated using the Taylor series method to adjust for stratification, weighting, and clustering of survey data. Multivariable significance tests in logistic regression analyses were made using Wald χ^2^ tests based on coefficient variance-covariance matrices adjusted for design effects using the Taylor series method.^43^ The AAS was augmented to increase coverage of soldiers stationed in Afghanistan and represented a clustered probability sample. The study used a stratified cluster design to account for time-space clustering of observations, and thus allowed for correct estimated variances and associated inferences about the study population.^22^ Stratification was defined by Army command and unit size within command. Clusters for the in-theater group were determined at the individual soldier level, as compared with those from the general AAS, which was sampled at the session level. Statistical significance was evaluated using 2-sided design-based tests and a .05 significance level. To address the potential for bias associated with the small number of cases (85), we compared parameter estimates from our final model with those based on a Firth penalized logistic regression model.^44^ To examine concentration of risk, we used the final model to generate estimated probabilities of SI and examined the proportion of cases among soldiers in the top ventile of estimated risk. Analyses were conducted using SAS statistical software version 9.4 (SAS Institute, Inc).

## Results

Of the 3957 soldiers in this study, 3473 (87.5%; all percentages weighted) were male and 2135 (52.6%) were aged 29 years or younger. Lifetime SI was reported by 512 soldiers (11.7%), with 32.0% of those reporting onset after enlistment. One hundred ninety-one soldiers (3.0%) reported past-year SI and 85 (1.9%; 95% CI, 1.4%-2.5%) reported past 30-day SI (2.0% of Regular Army; 1.7% of activated Army National Guard/Reserve soldiers). Among the 85 soldiers with 30-day SI, 42% reported onset after enlistment. Participants with 30-day SI were mostly male (81.3%), younger than 30 years (57.0%), white (82.2%), at least high school educated (88.7%), and married (57.0%). Similarly, soldiers without SI were mostly male (87.6%), younger than 30 (52.5%), white (67.1%), at least high school educated (91.8%), and married (58.9%) (Table 1).

**Table 1.** Association of Sociodemographic Characteristics With Past 30-Day Suicide Ideation Among Active-Duty US Army Soldiers During Deployment in Afghanistan

Characteristic	Univariable OR (95% CI)	Unweighted No. (Weighted %)		
		Suicide Ideation (n = 85)	No Suicide Ideation (n = 3872)	Total Population (N = 3957)
Sex				
Male	1 [Reference]	72 (81.3)	3401 (87.6)	3473 (87.5)
Female	1.5 (0.9-2.5)	13 (18.7)	471 (12.4)	484 (12.5)
χ^2^_1_	2.1			
Current age, y				
≤29	1.2 (0.8-1.9)	48 (57.0)	2087 (52.5)	2135 (52.6)
≥30	1 [Reference]	37 (43.0)	1785 (47.5)	1822 (47.4)
χ^2^_1_	0.6			
Race/ethnicity				
White	1 [Reference]	65 (82.2)	2542 (67.1)	2607 (67.4)
Other	0.4 (0.3-0.6)^a^	20 (17.8)	1330 (32.9)	1350 (32.6)
χ^2^_1_	16.7^a^			
Education				
<High school^b^	1.4 (0.6-3.2)	9 (11.3)	321 (8.2)	330 (8.2)
≥High school	1 [Reference]	76 (88.7)	3551 (91.8)	3627 (91.8)
χ^2^_1_	0.7			
Marital status				
Not married	1.1 (0.6-1.8)	33 (43.0)	1557 (41.1)	1590 (41.1)
Currently married	1 [Reference]	52 (57.0)	2315 (58.9)	2367 (58.9)
χ^2^_1_	0.1			

Abbreviation: OR, odds ratio.

^a^ Statistically significant at *P* < .05.

^b^ Less than high school includes general educational development credential, home study diploma, occupational program certificate, correspondence school diploma, high school certificate of attendance, adult education diploma, and other nontraditional high school credentials.

Soldiers reporting 30-day SI in theater were significantly more likely to make a documented SA (risk ratio, 43.70 [95% CI, 11.9-158.7]; χ^2^_1_ = 93.0; *P* < .001). Administrative records indicated that for the period between survey administration and 12 months after returning from deployment, 6% of soldiers with 30-day SI (5 participants) attempted suicide, with 2 attempting in theater (3%) and 3 in the initial 12 months after deployment (3.2%). Among the 3872 control soldiers, none attempted suicide in theater and 6 (0.14%) attempted within 12 months after deployment.

### Sociodemographic Characteristics and SI

In univariable analyses, only race/ethnicity was associated with 30-day SI (Table 1). When race/ethnicity and sex (included owing to its previous consistent association with SI) were examined together in a multivariable model, race/ethnicity remained significant, with SI less likely among nonwhite soldiers (Table 1).

### Stressors and SI

Separate exploratory factor analyses produced 3 lifetime stressor factors (combat trauma, noncombat trauma, and bullying or sexual assault) (eTable 1 in the Supplement) and 7 past 12-month factors (combat trauma, assault or injury to self or other, death or illness of a friend or family member, relationship problems, legal problems, accident, and being bullied by unit members) (eTable 2 in the Supplement). In univariable models, SI was associated with 2 lifetime factors (noncombat trauma; bullying or sexual assault) and five 12-month factors (assault or injury to self or other, death or illness of friend or family member, relationship problems, legal problems, and bullying by unit members) (Table 2).

**Table 2.** Associations of Stressful Events With Past 30-Day Suicide Ideation Among Active-Duty US Army Soldiers During Deployment in Afghanistan

Event	Univariable OR (95% CI)	Unweighted No. (Weighted %)		
		Suicide Ideation (n = 85)	No Suicide Ideation (n = 3872)	Total Population (N = 3957)
Lifetime stressful events^a^				
Combat trauma				
Yes	0.9 (0.5-1.6)	68 (77.9)	3139 (79.8)	3207 (79.8)
No	1 [Reference]	17 (22.1)	733 (20.2)	750 (20.2)
χ^2^_1_	0.1			
Noncombat trauma				
Yes	2.1 (1.1-3.9)^b^	71 (80.1)	2638 (65.7)	2709 (66.0)
No	1 [Reference]	14 (19.9)	1234 (34.3)	1248 (34.1)
χ^2^_1_	5.5^b^			
Bullying and sexual assault				
Yes	3.4 (1.9-6.3)^b^	28 (31.5)	482 (11.9)	510 (12.3)
No	1 [Reference]	57 (68.5)	3390 (88.1)	3447 (87.7)
χ^2^_1_	15.7^b^			
Past 12-mo stressful events^a^				
Combat trauma				
Yes	0.9 (0.5-1.6)	55 (62.4)	2535 (64.7)	2590 (64.7)
No	1 [Reference]	30 (37.6)	1337 (35.3)	1367 (35.3)
χ^2^_1_	0.1			
Assault or injury to self or other				
Yes	2.2 (1.4-3.7)^b^	40 (46.0)	1132 (27.5)	1172 (27.9)
No	1 [Reference]	45 (54.0)	2740 (72.5)	2785 (72.1)
χ^2^_1_	10.2^b^			
Death or illness of friend or family				
Yes	2.6 (1.5-4.4)^b^	55 (62.5)	1638 (39.4)	1693 (39.9)
No	1 [Reference]	30 (37.5)	2234 (60.6)	2264 (60.1)
χ^2^_1_	11.6^b^			
Relationship problems				
Yes	3.5 (2.1-5.8)^b^	40 (41.0)	707 (16.4)	747 (16.9)
No	1 [Reference]	45 (59.0)	3165 (83.6)	3210 (83.1)
χ^2^_1_	24.8^b^			
Legal problems				
Yes	4.1 (2.8-4.4)^b^	11 (12.6)	146 (3.4)	157 (3.6)
No	1 [Reference]	74 (87.4)	3726 (96.6)	3800 (96.4)
χ^2^_1_	14.9^b^			
Accident				
Yes	2.0 (0.7-5.5)	5 (6.5)	129 (3.4)	134 (3.4)
No	1 [Reference]	80 (93.5)	3743 (96.6)	3823 (96.6)
χ^2^_1_	1.8			
Bullied by unit members				
Yes	2.5 (1.2-5.4)^b^	11 (14.6)	251 (6.4)	262 (6.5)
No	1 [Reference]	74 (85.4)	3621 (93.6)	3695 (93.5)
χ^2^_1_	5.6^b^			

Abbreviation: OR, odds ratio.

^a^ Lifetime and past 12-month stressful event variables were derived from exploratory factor analyses. Variables indicate dichotomous endorsement of any event within a factor (yes or no) and cumulative scores of stressor factors.

^b^ Statistically significant at *P* < .05.

In multivariable models adjusting for sex and race/ethnicity, we examined dichotomous and continuous indicators for lifetime combat trauma, noncombat trauma, and bullying or sexual assault to determine the correct functional form of each factor. Continuous lifetime stressor variables significantly associated with SI were included in subsequent models; otherwise, the dichotomized form of the variable was included if significant. Lifetime combat trauma, noncombat trauma, and bullying or sexual assault were associated with increased odds of 30-day SI. Only the continuous score for lifetime noncombat trauma was significantly associated with SI. Examining the form of the association, this factor was subsequently dichotomized into levels indicating 0 to 6 vs 7 or more noncombat trauma events. A final lifetime stressor model, including significant combat trauma, bullying or sexual assault, and noncombat trauma factors indicated that bullying or sexual assault and noncombat trauma continued to be associated with SI (eTable 3 in the Supplement).

Similar analyses were conducted for past 12-month stressor variables. In multivariable models adjusting for sex and race/ethnicity, dichotomous relationship problems and legal problems factors and continuous combat trauma, assault or injury to self or other, and death or illness of friend or family member factors were significantly associated with SI. In a final model including these 5 factors, each remained associated with SI except for 12-month combat trauma (eTable 3 in the Supplement).

### MHDx and SI

Among all soldiers, 13.9% had at least one 30-day MHDx, 4.5% reported 30-day PTSD, and 4.1% had 30-day MDD (Table 3). Among soldiers with 30-day SI, 60.0% reported at least one 30-day MHDx, indicating that MHDx was more than 4 times more common in those with SI, with 37.7% reporting 2 or more disorders. In contrast, only 14.7% of soldiers without SI reported a 30-day MHDx. The most common 30-day MHDx among those with and without 30-day SI included PTSD (19.3% vs 4.2%), MDD (44.2% vs 3.3%), and IED (25.7% vs 6.4%) (Table 3). Among those who had 30-day SI with MDD, 63.0% also had PTSD, and 42.8% of those with PTSD had MDD.

**Table 3.** Association of Mental Disorders With Past 30-Day Suicide Ideation Among Active-Duty US Army Soldiers During Deployment in Afghanistan

Disorder	Univariable OR (95% CI)	Unweighted No. (Weighted %)		
		Suicide Ideation (n = 85)	No Suicide Ideation (n = 3872)	Total Population (N = 3957)
Internalizing disorders				
Major depressive disorder				
Past 30 d	39.7 (20.3-77.4)^a^	37 (44.2)	136 (3.3)	173 (4.1)
Prior to past 30 d	5.7 (2.9-11.0)^a^	28 (28.3)	647 (14.8)	675 (15.1)
No	1 [Reference]	20 (27.6)	3089 (81.9)	3109 (80.8)
χ^2^_2_	119.3^a^			
Bipolar disorder (lifetime)				
Yes	6.6 (2.8-15.3)^a^	9 (11.1)	91 (2.2)	100 (2.4)
No	1 [Reference]	76 (88.9)	3781 (97.8)	3857 (97.6)
χ^2^_1_				
Generalized anxiety disorder				
Past 30 d	12.2 (6.8-22.0)^a^	20 (22.4)	122 (3.1)	142 (3.5)
Prior to past 30 d	3.1 (1.8-5.6)^a^	29 (30.9)	702 (17.0)	731 (17.3)
No	1 [Reference]	36 (46.6)	3048 (79.9)	3084 (79.2)
χ^2^_2_	71.1^a^			
Panic disorder				
Past 30 d	7.7 (3.4-17.2)^a^	9 (11.6)	76 (1.7)	85 (1.8)
Prior to past 30 d	3.4 (0.7-16.3)	3 (2.6)	34 (0.9)	37 (0.9)
No	1 [Reference]	73 (86.3)	3762 (97.5)	3835 (97.3)
χ^2^_2_	29.6^a^			
Posttraumatic stress disorder				
Past 30 d	9.1 (4.7-17.9)^a^	18 (19.3)	172 (4.2)	190 (4.5)
Prior to past 30 d	3.8 (2.2-6.5)^a^	43 (44.2)	958 (23.4)	1001 (23.8)
No	1 [Reference]	24 (36.5)	2742 (72.4)	2766 (71.7)
χ^2^_2_	43.6^a^			
Externalizing disorders				
Substance use disorder				
Past 30 d	5.5 (1.2-25.3)^a^	3 (3.9)	30 (0.9)	33 (1.0)
Prior to past 30 d	3.9 (2.1-7.3)^a^	24 (27.8)	392 (9.5)	416 (9.8)
No	1 [Reference]	58 (68.3)	3450 (89.6)	3508 (89.2)
χ^2^_2_	20.5^a^			
Intermittent explosive disorder				
Past 30 d	5.4 (2.9-10.2)^a^	22 (25.7)	275 (6.4)	297 (6.7)
Prior to past 30 d	1.6 (0.8-3.1)	9 (10.6)	351 (8.8)	360 (8.8)
No	1 [Reference]	54 (63.7)	3246 (85.0)	3300 (84.5)
χ^2^_2_	27.8^a^			
Attention-deficit/hyperactivity disorder (past 6 mo)				
Yes	7.7 (3.6-16.6)^a^	14 (18.4)	112 (2.8)	126 (3.1)
No	1 [Reference]	71 (81.7)	3760 (97.2)	3831 (96.9)
χ^2^_1_	27.6^a^			

Abbreviation: OR, odds ratio.

^a^ Statistically significant at *P* < .05.

In univariable analyses, SI was associated with all MHDx. When examined together in a multivariable model adjusting for sex and race/ethnicity, only MDD remained significant (Table 3).

### Final Combined Models

When sex, race/ethnicity, and the significant lifetime and 12-month stressors were examined simultaneously, white race/ethnicity, lifetime noncombat trauma, relationship and legal problems, and death or illness of friend or family continued to be associated with SI (Table 4). Those 4 factors and sex were retained in a final model that also included MDD (the only MHDx associated with 30-day SI in previous analyses). White race/ethnicity (OR, 3.1 [95% CI, 1.8-5.1]), lifetime noncombat trauma (OR, 2.1 [95% CI, 1.1-4.0]), and MDD (past 30 days: OR, 31.8 [95% CI, 15.0-67.7]; prior to past 30 days: OR, 4.9 [95% CI, 2.5-9.6]) remained significantly associated with SI. Comparison of the final model with that found using Firth penalized logistic regression correction found identical ORs, suggesting no associated bias based on small sample size for cases. Using estimated probabilities from the final model, the 5% of soldiers in the top ventile of risk included 55.8% of participants with SI.

**Table 4.** Associations of Sociodemographic Characteristics, Stressful Events, and Mental Disorders With Past 30-Day Suicide Ideation Among Active-Duty US Army Soldiers During Deployment in Afghanistan

Factor	OR (95% CI)	
	Combined Multivariable Model 1^a^	Final Combined Multivariable Model 2^b^
Sociodemographic characteristics		
Sex		
Male	1 [Reference]	1 [Reference]
Female	1.5 (0.7-3.2)	1.5 (0.7-3.2)
χ^2^_1_	1.0	1.1
Race/ethnicity		
White	2.5 (1.6-3.8)^c^	3.1 (1.8-5.1)^c^
Other	1 [Reference]	1 [Reference]
χ^2^_1_	16.4^c^	19.2^c^
Lifetime stressful events^d^		
Bullying and sexual assault^e^		
Yes	1.9 (1.0-3.9)	
No	1 [Reference]	
χ^2^_1_	3.5	
Noncombat trauma^f^		
≥7 events	2.2 (1.3-3.6)^c^	2.1 (1.1-4.0)^c^
0-6 events	1 [Reference]	1 [Reference]
χ^2^_1_	9.2^c^	5.4^c^
Past 12-mo stressful events^d^		
Relationship problems^e^		
Yes	2.2 (1.3-3.8)^c^	1.5 (0.8-2.7)
No	1 [Reference]	1 [Reference]
χ^2^_1_	9.7^c^	1.5
Legal problems^e^		
Yes	2.3 (1.1-5.0)^c^	1.7 (0.7-4.5)
No	1 [Reference]	1 [Reference]
χ^2^_1_	4.5^c^	1.3
Assault or injury of self or other^f^		
≥4 events	2.2 (1.0-5.3)	
0-3 events	1 [Reference]	
χ^2^_1_	3.6	
Death or illness of friend or family^f^		
≥3 events	1.9 (1.0-3.4)^c^	1.2 (0.6-2.3)
0-2 events	1 [Reference]	1 [Reference]
χ^2^_1_	4.6^c^	0.2
Mental disorders		
Major depressive disorder		
Past 30 d		31.8 (15.0-67.7)^c^
Prior to past 30 d		4.9 (2.5-9.6)^c^
No		1 [Reference]
χ^2^_1_		81.7^c^

Abbreviation: OR, odds ratio.

^a^ Model includes significant multivariable sociodemographic and lifetime and past 12-month dichotomous and cumulative stressor factors from Table 1 and eTable 3 in the Supplement. Sex, while nonsignificant in the univariable model, was included because of its consistent association with suicide ideation in previous Army studies.

^b^ Model includes significant multivariable sociodemographic, stressor, and mental disorder factors from Table 1, Table 2, and eTable 3 in the Supplement.

^c^ Statistically significant at *P* < .05.

^d^ Lifetime and past 12-month stressful event variables were derived from exploratory factor analyses.

^e^ Variables indicate dichotomous endorsement of any event within a factor (yes or no).

^f^ Variable indicates cumulative score of the number of endorsed items within the factor.

## Discussion

This study provides a rare examination of 30-day SI and MH burden during combat deployment in a representative sample of soldiers at middeployment. Importantly, middeployment has been identified as the highest risk period for SA among soldiers in theater.^5,7^ Approximately 14% of middeployment soldiers had a current MHDx (eg, 4.5% with PTSD and 4.1% with MDD), a rate similar to that found for in-theater soldiers in previous research conducted by the military.^10^ In our study, 3.0% of middeployment soldiers reported past-year SI and 1.9% reported past 30-day SI. Soldiers reporting 30-day SI at middeployment were nearly 44 times more likely than those without SI to subsequently attempt suicide through the first year after returning from deployment. Screening for SI in deployed soldiers may be an effective strategy for identifying those at risk of SA. Increasing our understanding of factors associated with the transition to SA among individuals with SI, and whether identification of SI in theater differs from that in noncombat environments, warrants further research.

Substantial middeployment MH burden was observed among those with current SI, with 60% having at least 1 MHDx. However, 40% of soldiers with SI did not report any current MHDx. This may reflect subthreshold or underreported MHDx, as observed in previous studies^45,46,47^ of suicidal behaviors among soldiers and civilians. Importantly, these soldiers would not be identified through usual MHDx screening. Use of more sensitive measures of subthreshold diagnosis that focus on MH problems at the symptom level may aid in better capturing psychological distress.

Rates of MHDx were substantially higher among soldiers with 30-day SI compared with those without SI (eg, MDD [44.2% vs 3.3%] and IED [25.7% vs 6.4%]). Current SI was also associated with most lifetime and 12-month stressors. The high rate of IED associated with suicidal behaviors has been previously observed in soldiers.^18,36^ In this study, the high IED rate may reflect the frequency of anger-related symptoms among deployed soldiers. The association of anger and depression with SI among deployed soldiers warrants further study.

Although PTSD was a significant univariable factor associated with SI, only MDD remained significant when all MHDx were examined together, suggesting the comorbidity of MDD and PTSD and the unique associations of MDD with SI during deployment. Identifying the specific dimensions of MDD associated with SI may aid in understanding the mechanisms influencing this association. Previous veteran studies suggest that specific cognitive-affective symptoms (eg, depressed mood, excessive or inappropriate guilt) are associated with SI.^48^ However, it is not known whether this same pattern of MDD symptoms is associated with risk among deployed soldiers.

In this sample of deployed soldiers, sex was not associated with 30-day SI. Previous studies found that female soldiers had higher odds of pre-enlistment SI (OR 1.4)^38^ and higher lifetime SI rates (20.1%) compared with male soldiers (12.7%).^49^ The absence of an association between sex and SI during deployment may be the result of the small number of women in the sample, the selection process for deployment, or currently unknown sex-specific changes in SI risk during deployment.

The final multivariable model indicated the particular importance of lifetime trauma and MDD to SI during deployment. A similar association of lifetime trauma with SI has been reported in nondeployed soldiers and veterans.^12,50,51,52^ However, the mechanisms influencing this association are complex. At time of entry into the Army, 74% of new soldiers report previous trauma exposure,^53^ with one-fifth indicating childhood maltreatment.^50^ Soldiers reporting childhood adversity have higher SI rates before Army service^50^ and are at greater risk of MDD and generalized anxiety disorder when exposed to high stress.^54^ Identification of the mediators that may influence the association between lifetime trauma and SI in larger samples of deployed soldiers is needed.

Although previous studies^18,36,49,55^ of military populations have examined similar risk factors for suicidal behaviors, primarily sociodemographic characteristics and MHDx, as observed in this study, investigation of different outcomes (eg, SI vs SA) using different samples (eg, deployed soldiers only vs all active-duty soldiers, Regular Army vs National Guard/Reserve, and veterans) may complicate comparison and not fully capture the experience of recent SI, specifically during deployment. These differences are most clearly noted in the absence of associations of several sociodemographic characteristics with 30-day SI in our study. However, the consistency of findings across studies for MHDx, specifically MDD, and race/ethnicity as factors associated with lifetime and current suicidal behaviors, is important and merits further study.

### Limitations

Our results should be interpreted with certain limitations. Self-report data are subject to recall bias. Our sample is representative of deployed soldiers in Afghanistan at the time of the survey and may not be generalizable to soldiers deployed during other phases of the war, other military populations, veterans, or civilians. Soldiers may have underreported SI or MHDx owing to concerns about confidentiality or stigma.^56^ We used screeners rather than full diagnostic interviews to assess MHDx.^22^ Capture of SAs is limited to those entered in administrative records, and may miss SAs that did not receive clinical intervention. Further study of attempts using self-report measures would increase identification of SAs that may not have received medical attention. Furthermore, as this study focused only on active-duty soldiers during deployment, the patterns documented may not continue after deployment or after separation.

## Conclusions

This survey study found increased odds of SA in deployed soldiers with 30-day SI at middeployment. A substantial proportion of deployed soldiers with SI did not report any MHDx. Developing specific outreach efforts for these at-risk soldiers who do not meet MHDx criteria can aid in identification of soldiers at risk of suicide. Lifetime noncombat trauma and MDD are important factors associated with SI among deployed soldiers. Research examining deployment experiences that increase SI in soldiers with past trauma and MDD may assist clinicians and leadership in identifying and treating those at increased risk. Understanding how these factors may change at various times during deployment could aid in understanding how the middeployment period is associated with SI.

## Notes

Supplement. **eMethods. **Sample, Measures, and Statistical Analysis **eTable 1. **Rotated Factor Loadings of Lifetime Stressful Event Items in U.S. Army Soldiers Deployed in Afghanistan (n=3,957) **eTable 2. **Rotated Factor Loadings of Past 12-Month Stressful Event Items in U.S. Army Soldiers Deployed in Afghanistan (n=3,957) **eTable 3. **Associations of Lifetime and Past 12-Month Stressful Events With 30-day Suicide Ideation in U.S. Army Soldiers Deployed in Afghanistan
