# Supplementary material for: Factors Associated With Suicide Ideation in US Army Soldiers During Deployment in Afghanistan
Source: JAMA Netw Open. 2020 Jan 29;3(1):e1919935. doi: 10.1001/jamanetworkopen.2019.19935 (PMC6991281; doi:10.1001/jamanetworkopen.2019.19935)
Supplement: Supplement. — eMethods. Sample, Measures, and Statistical Analysis eTable 1. Rotated Factor Loadings of Lifetime Stressful Event Items in U.S. Army Soldiers Deployed in Afghanistan (n=3,957) eTable 2. Rotated Factor Loadings of Past 12-Month Stressful Event Items in U.S. Army Soldiers Deployed in Afghanistan (n=3,957) eTable 3. Associations of Lifetime and Past 12-Month Stressful Events With 30-day Suicide Ideation in U.S. Army Soldiers Deployed in Afghanistan [file jamanetwopen-3-e1919935-s001.pdf]

Supplementary Online Content

Ursano RJ, Herberman Mash HB, Kessler RC, et al. Factors associated with suicide ideation in US Army soldiers during deployment in Afghanistan. *JAMA Netw Open*. 2020;3(1):e1919935. doi:10.1001/jamanetworkopen.2019.19935

eMethods. Sample, Measures, and Statistical Analysis
eTable 1. Rotated Factor Loadings of Lifetime Stressful Event Items in U.S. Army Soldiers Deployed in Afghanistan (n=3,957)
eTable 2. Rotated Factor Loadings of Past 12-Month Stressful Event Items in U.S. Army Soldiers Deployed in Afghanistan (n=3,957)
eTable 3. Associations of Lifetime and Past 12-Month Stressful Events With 30-day Suicide Ideation in U.S. Army Soldiers Deployed in Afghanistan

This supplementary material has been provided by the authors to give readers additional information about their work.

© 2020 Ursano RJ et al. *JAMA Network Open.*

eMETHODS: Sample, Measures, and Statistical Analysis

Sample

The current study was based on a sample of soldiers deployed in Afghanistan in support of Operation Enduring Freedom in July 2012. The data are from self-administered questionnaires (SAQs) collected as part of the All Army Study (AAS),[20-22] a representative survey of active duty soldiers (including Regular Army and activated Army National Guard and Army Reserve) serving inside and outside the continental U.S, exclusive of those in Initial Military Training. Constraints on survey administration in Afghanistan resulted in data collection in Kuwait as soldiers were transiting for mid-deployment leave.

Among the soldiers deployed in Afghanistan who were recruited for the study, 80.9% provided informed consent, and 86.5% of these soldiers completed the SAQ. Of those who completed the SAQ, 55.6% consented to linkage of their SAQ responses to Army and Department of Defense administrative records, totaling 3,987 in-theater respondents.[18] After removal of 30 respondents due to: 1)unverified Army status (*n*=3); 2)administration of the incorrect version of the mid-deployment SAQ (*n*=6); and/or 3)incorrect identification of soldiers' deployment status (*n*=21), the final sample included 3,387 soldiers who were transitioning to their mid-deployment leave (outbound) and 570 transitioning from their leave (inbound), providing an important snapshot at mid-deployment.

The sample was weighted to be representative of all active duty soldiers serving in Afghanistan in July 2012 (final weighted sample *N*=87,032). We obtained de-identified administrative data for the entire Army and for survey respondents who agreed to administrative data linkage, allowing two weights to be created to adjust for nonresponse bias (i.e., discrepancies between the analytic sample and target population). Each weight was constructed based on an iterative process of stepwise logistic regression analysis designed to obtain a stable weighting solution. Weight 1 (W1) adjusted for

© 2020 Ursano RJ et al. *JAMA Network Open.*

discrepancies between survey completers with and without administrative record linkage based on a prediction equation using SAQ responses as predictors: W1 = 1/p1, where p1 is the probability of consenting to administrative data linkage. Weight 2 (W2) adjusted for discrepancies between weighted (W1) survey completers with record linkage and the target population based on a prediction equation using a small set of administrative variables as predictors (e.g., age, sex, rank): W2 = 1/p2, where p2 is the probability of survey completion. These doubly weighted (W1 × W2) data were used in the current study's analyses (additional detailed information regarding the weighting process, which allows for report of population prevalence estimates, in available in Kessler et al.[22]).

Measures

Socio-demographic characteristics. Army and Department of Defense administrative personnel records were used to construct socio-demographic variables (gender, age, race/ethnicity, education, and marital status).

MHDx. The SAQ included self-administered assessment of DSM-IV internalizing and externalizing disorders. The Composite International Diagnostic Interview screening scales (CIDI-SC)[23,24] assessed past 30-day MDD, generalized anxiety disorder (GAD), panic disorder (PD), substance use disorder (SUD; alcohol or drug abuse or dependence, including illicit drugs and misused prescription drugs), and intermittent explosive disorder (IED), and past 6-month attention-deficit/hyperactivity disorder (ADHD). Past 30-day PTSD was assessed using the PTSD Checklist (PCL).[25] The CIDI-SC also assessed lifetime PD, IED, and bipolar disorder I-II or sub-threshold bipolar disorder (BPD). Sub-threshold BPD was defined as lifetime history of hypomania without MDD history, or sub-threshold hypomania with history of MDD.[26] Lifetime MDD, GAD, SUD, and PTSD were assessed using a revised self-administered version of the Family History Screen (FHS),[27] modified to assess personal, rather than family, MHDx history.

© 2020 Ursano RJ et al. *JAMA Network Open.*

All disorders were assessed without DSM-IV diagnostic hierarchy or organic exclusion rules. The CIDI-SC and PCL have good concordance with independent clinical diagnoses in the AAS (area under the receiver operating characteristic curve of 0.69-0.79 across diagnoses).[28] The FHS has acceptable concordance with best-estimate clinical diagnoses,[27] although the items used in the AAS yielded implausibly high prevalence estimates, and FHS diagnoses should consequently be considered combinations of threshold and sub-threshold disorders. The MHDx data were used to construct recency variables (past 30 days, prior to past 30 days, no diagnosis) for MDD, GAD, PD, PTSD, SUD, and IED. BP was examined only as a lifetime diagnosis (due to small N's) and ADHD was examined only as a past 6-month diagnosis (due to how it was assessed).

Stressors. We assessed lifetime and past 12-month exposure to traumatic and stressful events. Using items from the Joint-Mental Health Advisory Team 7 (J-MHAT 7)[29] and Deployment Risk and Resilience Inventory (DRRI),[30] respondents were asked how many times they had experienced 15 deployment-related stressors (e.g., fired rounds at the enemy or taken enemy fire, wounded by enemy, members of unit seriously wounded or killed, hazed or bullied by unit members) and 14 life stressors excluding deployment experiences (e.g., serious physical assault, sexual assault or rape, murder of close friend or relative, life-threatening illness/injury, disaster). An item inquiring about childhood or adolescent bullying was excluded from the past 12-month stressors due to the timeframe of the question. Responses to these questions were dichotomized (yes/no). Respondents also indicated (yes/no) whether they had experienced any of the events in the past 12 months. Additional 12-month stressors were assessed (yes/no) using 16 items from the Life Events Questionnaire[31] and 2008 Department of Defense Survey of Health-Related Behaviors among Active Duty Military Personnel[32] (e.g., life-threatening illness

of close friend/family member, separation or divorce, caused an accident where someone else was hurt, trouble with police).

Suicide ideation and attempts. Past 30-day SI was assessed using a modified version of the Columbia Suicidal Severity Rating Scale.[33] Respondents endorsing lifetime SI ("Did you ever in your life have thoughts of killing yourself?" or "Did you ever wish you were dead or would go to sleep and never wake up?") were then asked whether they had those thoughts in the past 30 days and the age at which they last experienced SI. Administratively documented SAs in-theater and up to 12 months post-deployment were identified using records from the DoD Suicide Event Report (DoDSER),[34] and ICD-9-CM diagnostic codes E950-E958 from the Military Health System Data Repository, Theater Medical Data Store, and TRANSCOM (Transportation Command) Regulating and Command and Control Evacuating System (TRAC2 ES).[35]

Statistical Analysis

MHDx frequencies were calculated. Past 30-day SI predictors were examined in stages. We first examined the univariable associations of socio-demographic variables with ideation. Socio-demographic variables significant at the univariable level were examined together in a multivariable model. A similar process was used for MHDx variables and included significant socio-demographic variables identified in the first stage of analysis.

Due to the small number of cases and large number of stressor items, exploratory factor analysis (EFA) was used as a data reduction method to identify latent stressor subgroups. We conducted a polychoric EFA with Promax rotation using the lifetime stressors (30 items), followed by a similar EFA using past 12-month stressors (46 items). Missing values were imputed to "no". The number of factors was determined based on eigenvalues ≥1 and scree plot

© 2020 Ursano RJ et al. *JAMA Network Open.*

examination. Items were assigned to factors based on loadings ≥0.40. Cross-loading items were assigned to the factor on which they loaded highest. A cumulative score was generated for each factor by summing the number of endorsed items. Dichotomous variables were also created to indicate any stressor exposure within a given factor. Associations of these stressor variables with SI were examined in a series of logistic regression models. The factor-based, dichotomous lifetime stressor variables were first examined together in a multivariable model, followed by a separate model that also included the cumulative scores for each lifetime factor. A final lifetime stressor model including any significant cumulative score factors, together with any significant dichotomous factors that were not significantly associated with SI at the cumulative level, was conducted. This procedure was repeated with the factor-based 12-month stressors. Significant stressor variables from the final lifetime and past 12-month stressor models were then included together in a combined model predicting SI. This approach of testing the contribution of an entire set of variables with a multiple degrees of freedom test addresses possible correlations among the variables and improves model selection.[42]

Significant variables from the socio-demographic, stressor, and MHDx analyses were then examined together in combined models. Logistic regression coefficients were exponentiated to obtain odds-ratios (OR) and 95% confidence intervals (CI). Standard errors were estimated using the Taylor series method to adjust for stratification, weighting, and clustering of survey data. Multivariable significance tests in logistic regression analyses were made using Wald χ^2 tests based on coefficient variance-covariance matrices adjusted for design effects using the Taylor series method.[43] Statistical significance was evaluated using two-sided design-based tests and a 0.05 significance level. To examine concentration of risk, we used the final model to generate predicted probabilities of SI. Those probabilities were sorted into

© 2020 Ursano RJ et al. *JAMA Network Open.*

ventiles and we examined the proportion of cases among soldiers in the top ventile of predicted risk.

© 2020 Ursano RJ et al. *JAMA Network Open.*

eTable 1. Rotated factor loadings of lifetime stressful event items in U.S. Army soldiers deployed in Afghanistan (*n*=3,957).[1]				
	**Rotated factor pattern (standardized regression coefficients)**			
**Lifetime stressor items**	**Factor 1**	**Factor 2**	**Factor 3**	**Factor name**
Have direct responsibility for the death of US or ally personnel (D)	0.923			**Combat trauma**
Have direct responsibility for the death of an enemy combatant (D)	0.918			
Have direct responsibility for the death of a non-combatant (D)	0.908			
Get wounded by the enemy (D)	0.892			
Have a close call (D)	0.857			
Get exposed to the sights, sounds, or smells of severely wounded or dying people or see dead bodies (D)	0.851			
Go on combat patrols or have other dangerous duty (D)	0.846			
Fire rounds at enemy or take enemy fire (D)	0.823			
You were sexually assaulted or raped (D)	0.797			
You were seriously physically assaulted (e.g., mugged) (D)	0.776			
Save the life of a Soldier or a civilian (D)	0.770			
Witness violence within the local population or mistreatment toward non-combatants (D)	0.767			
See homes or villages that had been destroyed or people begging for food (D)	0.762			
Have member(s) of your unit/platoon who were seriously wounded or killed (D)	0.744			
Serious assault of a friend or relative		0.778		**Non-combat trauma**
Close loved one at risk of death or injury		0.758		
Bullied during childhood or adolescence		0.691		
Attempted suicide of a friend or relative		0.689		
Serious physical assault		0.657		
Suicide of a friend or relative		0.654		
Other experience		0.642		
Accidental death of a friend or relative		0.614		
Murder of a friend or relative		0.587		
Witnessed someone being injured or killed		0.545		
Life-threatening illness		0.532		
In a disaster		0.524		
Discovered or handled dead bodies		0.463		
Combat death of a friend or relative		0.421		
You were bullied by one or more members of your unit (D)			0.697	**Bullying and sexual assault**
Serious sexual assault or rape			0.590	

© 2020 Ursano RJ et al. *JAMA Network Open.*

**Eigenvalue**	12.27	4.63	2.12	
**Variance explained by each factor eliminating other factors**	9.50	4.90	2.34	
**Percent of total variance[2]**	10.81	5.88	2.34	

[1]Based on exploratory factor analysis with polychoric correlations and Promax rotation. Number of factors was determined based on eigenvalues $\geq$ 1. Items were assigned to factors based on factor loadings $\geq$ 0.4. In cases of cross-loading, the factor with the higher loading was chosen.
[2]Obtained by dividing the "Variance Explained by Each Factor Eliminating Other Factors" by the total number of items.
(D) = Items assessed as deployment-related stressful events.

© 2020 Ursano RJ et al. *JAMA Network Open.*

eTable 2. Rotated factor loadings of past 12-month stressful event items in U.S. Army soldiers deployed in Afghanistan (*n*=3,957).								
	**Rotated factor pattern (standardized regression coefficients)**							
**Past 12-month stressor items**	**Factor 1**	**Factor 2**	**Factor 3**	**Factor 4**	**Factor 5**	**Factor 6**	**Factor 7**	**Factor name**
Go on combat patrols or have other dangerous duty (D)	0.958							**Combat trauma**
Get exposed to the sights, sounds, or smells of severely wounded or dying people or see dead bodies (D)	0.931							
Fire rounds at enemy or take enemy fire (D)	0.926							
Have direct responsibility for the death of an enemy combatant (D)	0.906							
See homes or villages that had been destroyed or people begging for food (D)	0.883							
Have a close call (D)	0.876							
Witness violence within the local population or mistreatment toward non-combatants (D)	0.863							
Have direct responsibility for the death of US or ally personnel (D)	0.837							
Have direct responsibility for the death of a non-combatant (D)	0.832							
Get wounded by the enemy (D)	0.807							
Save the life of a Soldier or a civilian (D)	0.792							
You were seriously physically assaulted (e.g., mugged) (D)	0.774							
Have member(s) of your unit/platoon who were seriously wounded or killed (D)	0.761							
You were sexually assaulted or raped (D)	0.736							
Serious physical assault		0.964						**Assault or injury of self/other**
Serious sexual assault or rape		0.935						
In a disaster		0.935						
Attempted suicide of a friend or relative		0.927						
Murder of a friend or relative		0.909						
Life-threatening illness		0.909						
Suicide of a friend or relative		0.843						
Serious assault of a friend or relative		0.803						
Accidental death of a friend or relative		0.800						
Close loved one at risk of death or injury		0.593						
Combat death of a friend or relative		0.513						
Other experience		0.510						
Witnessed someone being injured or killed		0.476						
Discovered or handled dead bodies		0.437						
Death of a friend or family member			0.635					**Death/illness of friend/family**
Life-threatening illness of a friend or family member			0.605					
Any other stressful event			0.573					
Arguments with someone in unit			0.569					
Any other serious legal problem			0.518					

© 2020 Ursano RJ et al. *JAMA Network Open.*

Did not get promoted when you should have			0.424					
Got a lower score than you expected on an efficiency report			0.383					
Spouse or partner cheated on you				0.906				**Relationship problems**
Separation or divorce				0.865				
Betrayal by someone else close to you				0.774				
Ongoing arguments or break-up with friend or family member				0.609				
Spent time in jail, stockade, correctional custody, brig					0.930			**Legal problems**
Had trouble with the police					0.798			
Received UCMJ punishment					0.535			
Involved in a motor vehicle accident while you were driving						0.903		**Accident**
Caused an accident where someone else was hurt						0.753		
You were bullied by one or more members of your unit (D)							0.649	**Bullied by unit**
**Eigenvalue**	18.03	5.90	3.77	2.17	1.70	1.24	1.02	
**Variance explained by each factor eliminating other factors**	7.40	5.56	2.03	2.26	2.03	1.53	1.61	
**Percent of total variance[2]**	11.79	9.15	3.35	3.27	2.74	1.91	1.63	

[1]Based on exploratory factor analysis with polychoric correlations and Promax rotation. Number of factors was determined based on eigenvalues ≥ 1. Items were assigned to factors based on factor loadings ≥ 0.4. In cases of cross-loading, the factor with the higher loading was chosen.

[2]Obtained by dividing the "Variance Explained by Each Factor Eliminating Other Factors" by the total number of items.

(D) = Items assessed as deployment-related stressful events.

© 2020 Ursano RJ et al. *JAMA Network Open.*

eTable 3. Associations of lifetime and past 12-month stressful events with 30-day suicide ideation in U.S. Army soldiers deployed in Afghanistan.

	Predictors of past 30-day suicide ideation[1] (*n*=85 cases, *n*=3,872 controls)			
	Multivariable 1[2]		Multivariable 2[3]	
	**OR**	**(95% CI)**	**OR**	**(95% CI)**
**Lifetime stressful events[4]**				
Combat trauma				
Any				
None				
χ^2_1				
Non-combat trauma				
7+ events	3.3*	(1.9–5.9)		
0-6 events	1.0	–		
χ^2_1	17.5			
Bullying and sexual assault				
Any	2.7*	(1.3–5.5)		
None	1.0	–		
χ^2_1	7.8*			
**Past 12-month stressful events[4]**				
Combat trauma				
0-4 events				
5-6 events				
7+ events				
χ^2_1				
Assault or injury of self/other				
4+ events			3.1*	(1.3-7.3)
0-3 events			1.0	–
χ^2_1			6.8*	
Death/illness of friend/family				
3+ events			2.4*	(1.3-4.5)
0-2 events			1.0	–
χ^2_1			8.4*	
Relationship problems				
Any			2.6*	(1.5-4.3)
None			1.0	–
χ^2_1			12.8*	
Legal problems				
Any			2.4*	(1.1-5.3)
None			1.0	–
χ^2_1			4.4*	
Accident				
Any				
None				
χ^2_1				
Bullied by unit members				
Any				
None				
χ^2_1				

[1]Data were weighted to be representative of soldiers serving in Afghanistan during July 2012.
[2]Model includes all significant lifetime stressful event variables, plus gender and race (not shown).
[3]Model includes all significant past 12-month stressful event variables, plus gender and race (not shown).
[4]Lifetime and past-12-month stressful event variables were derived from exploratory factor analyses. Variables indicate either endorsement of any event within a factor (any/none), or are dichotomous categories based on the sums of the number of endorsed items within each factor.
*$p < 0.05$.

© 2020 Ursano RJ et al. *JAMA Network Open.*
